# Strain-associated variation in extracellular chemical profiles of fruit-derived *Lactiplantibacillus plantarum* under standardized cultivation

**DOI:** 10.3389/fmicb.2026.1966445

**Published:** 2026-10-30

**Authors:** Gabriela N. Tenea, Victor Cifuentes

**Affiliations:** Biofood and Nutraceutics Research and Development Group, Faculty of Engineering in Agricultural and Environmental Sciences, Universidad Técnica del Norte, Ibarra, Ecuador

**Keywords:** cell-free supernatants, extracellular chemical profile, *lactiplantibacillus plantarum*, LC-MS/MS, strain-level variation, untargeted metabolomics

## Abstract

**Introduction:**

*Lactiplantibacillus plantarum* exhibits substantial strain-level metabolic diversity, yet comparative information on extracellular chemical profiles among fruit-derived strains remains limited.

**Methods:**

This study characterized and compared cell-free supernatants from five native fruit-derived *L. plantarum* strains cultivated under standardized conditions, using three independent biological cultures per strain. Untargeted LC–MS/MS profiling using RP-HSS T3 chromatography in positive- and negative-ion electrospray modes and HILIC-BEH Amide chromatography in negative-ion mode yielded 8,692 analytical detections, of which 6,769 had associated MS/MS information. Following annotation-confidence filtering, quality-control assessment, and consolidation across analytical configurations, 999 higher-confidence metabolite annotations (Levels 1a and 1b) were retained for comparative analysis.

**Results:**

Multivariate analyses revealed strain-associated differences in extracellular chemical profiles, with the extent of separation varying among strains. Welch's one-way ANOVA followed by Benjamini–Hochberg correction identified 438 of the 999 higher-confidence annotations as significantly different among strains (FDR < 0.05). Pairwise Welch's *t*-tests with FDR correction and |log_2_FC| ≥ 1 revealed strongly comparison-dependent differentiation; among comparisons centered on G1, 4, 39, 62, and 18 FDR-supported annotations were identified for G1 vs. G2, G1 vs. G3, G1 vs. G4, and G1 vs. G5, respectively. Comparison with an incubated uninoculated MRS control showed that part of the extracellular chemical background was also detectable in the cultivation medium; consequently, compounds detected in the cell-free supernatants were not attributed exclusively to bacterial biosynthesis.

**Conclusion:**

These findings demonstrate substantial strain-associated variation in the extracellular chemical profiles of fruit-derived *L. plantarum* under standardized cultivation conditions and show that extracellular differentiation involves comparison-specific combinations of metabolite annotations rather than a uniform discriminatory signature. The higher-confidence candidates identified provide a basis for subsequent targeted quantitative and functional investigation of strain-associated extracellular phenotypes.

## 1 Introduction

*Lactiplantibacillus plantarum* (formerly *Lactobacillus plantarum*) is one of the most metabolically versatile species within the lactic acid bacteria (LAB), owing to its capacity to inhabit and adapt to diverse ecological niches. The species is naturally associated with fermented foods, plant materials, and the gastrointestinal tract of humans and animals, reflecting its ability to grow and persist under distinct environmental conditions (Fidanza et al., 2021; Garcia-Gonzalez et al., 2021; Martino et al., 2016; Yilmaz et al., 2022). Its relatively large genome, generally ranging from approximately 3.0 to 3.3 Mb, contains an extensive repertoire of genes involved in carbohydrate utilization, stress responses, transport, and regulation, providing a genetic basis for adaptation to heterogeneous environments (Garcia-Gonzalez et al., 2022). This genomic flexibility is associated with considerable metabolic diversity and with the capacity to produce, modify, transport, or release compounds including organic acids, exopolysaccharides, bacteriocins, vitamins, amino acid derivatives, and other metabolites (Zhang et al., 2025).

Fruit-associated environments are complex microbial habitats characterized by variation in pH, nutrient availability, osmotic conditions, oxidative stress, and phytochemical composition. Microorganisms inhabiting these environments encounter diverse carbon and nitrogen sources and other environmental pressures that can influence microbial growth and metabolic activity (Filannino et al., 2018; Tenea et al., 2025). Consistent with this ecological diversity, considerable genetic and phenotypic variation has been reported among naturally occurring *L. plantarum* populations (Tenea, 2022; Tenea et al., 2026). Isolates recovered from different plant-associated environments therefore provide a relevant biological resource for examining the extent of metabolic variation within this species. However, differences observed after cultivation under standardized laboratory conditions should be interpreted as strain-associated phenotypic variation rather than as direct evidence of adaptation to the original fruit host.

Metabolomic profiling provides a useful approach for characterizing phenotypic variation by examining differences in small-molecule composition among bacterial strains (Molina et al., 2025). In extracellular metabolomics, however, the detected chemical profile reflects not only compounds released or accumulated by microbial cells but also residual culture-medium components and compounds resulting from microbial substrate utilization or transformation, particularly when chemically complex growth media are used (Kaewnongkae et al., 2026; Liu L. et al., 2025). High-resolution liquid chromatography–tandem mass spectrometry (LC–MS/MS) provides broad chemical coverage and is particularly suitable for profiling diverse extracellular compounds, including organic acids, amino acid derivatives, peptides, lipids, carbohydrates, and other molecules associated with bacterial growth and metabolism (Aarika et al., 2025; Kaewnongkae et al., 2026). Comparative profiling under standardized cultivation conditions can therefore be used to examine strain-associated differences in extracellular chemical composition while controlling major sources of experimental variation such as cultivation medium, temperature, and incubation time.

Recent studies support the value of this approach for distinguishing extracellular metabolic phenotypes among LAB and other probiotic bacteria. Lu et al. (2026) used untargeted metabolomics to compare the secretomes of *Lacticaseibacillus rhamnosus* R0011 and *Bifidobacterium longum* R0175 and identified distinct extracellular profiles involving branched-chain hydroxy and aromatic lactic acids, amino acid derivatives, organic acids, vitamins, and other metabolites. Importantly, comparison with the corresponding medium showed both depletion and accumulation of specific compounds during bacterial growth, illustrating the contribution of substrate utilization and metabolic transformation to the final extracellular chemical profile. Similarly, untargeted metabolomic analysis of four *Bifidobacterium* strains revealed substantial strain-dependent metabolic variability (Liu K. et al., 2025). These studies illustrate the value of comparative metabolomics for characterizing extracellular chemical variation under controlled cultivation conditions.

Despite the recognized metabolic versatility of *L. plantarum*, comparative information on extracellular metabolomic variation among naturally occurring strains remains limited. Previous studies have demonstrated substantial metabolic heterogeneity within *L. plantarum*, but systematic comparisons of extracellular chemical profiles among multiple native strains cultivated under the same experimental conditions remain scarce. Characterizing this variation may improve understanding of the metabolic heterogeneity occurring within the species and provide a basis for prioritizing extracellular metabolite candidates for subsequent targeted investigation.

Previous studies from our group characterized individual strains included in the present work in terms of genomic features, probiotic-associated traits, antimicrobial activity, and integrated genomic and metabolomic characteristics (Molina et al., 2025; Tenea et al., 2025, 2026). These studies provided evidence of functional and metabolic diversity among fruit-derived *L. plantarum* strains but focused on different biological questions. A comparative assessment of their extracellular chemical profiles under standardized experimental conditions has not yet been performed.

Accordingly, this study aimed to characterize and compare the extracellular chemical profiles of five native fruit-derived *L. plantarum* strains using untargeted LC–MS/MS metabolomics. The analysis focused on strain-associated differences in extracellular chemical composition and relative metabolite abundance, as well as on the identification of higher-confidence metabolite annotations associated with the observed variation. This comparative approach provides insight into extracellular chemical diversity among *L. plantarum* strains and identifies candidate metabolites for further targeted investigation.

## 2 Materials and methods

### 2.1 Bacterial strains and preparation of CFS

Five native *L. plantarum* strains isolated from different fruit hosts in Ecuador were included in this study. The strains comprised UTNGt2 and UTNGt5, isolated from *Theobroma grandiflorum*; UTNGt3, isolated from *Chrysophyllum oliviforme*; UTNGt21A, isolated from *Solanum quitoense*; and UTNGt28L, isolated from *Chrysophyllum cainito*. Strains were maintained as frozen stock cultures in de Man, Rogosa and Sharpe (MRS) broth (Difco, Detroit, MI, USA). Three independent biological cultures were prepared for each strain, resulting in 15 biological samples (Table S1). Each culture originated from an independent inoculation and was processed independently throughout cultivation, cell-free supernatant (CFS) preparation, sample extraction, liquid chromatography–tandem mass spectrometry (LC–MS/MS) acquisition, and subsequent data analysis. For CFS preparation, bacterial cultures were inoculated into fresh MRS broth and incubated at 37 °C for 24 h. Growth was monitored spectrophotometrically at 600 nm (OD_600_) (Figure S1). The spectrophotometer had an upper reportable OD_600_ value of 3.0; consequently, measurements reaching this value represent the instrumental upper limit and were not interpreted as evidence of identical biomass among cultures. At the 24-h CFS collection point, viable counts ranged from 2.72 × 10^9^ to 3.08 × 10^9^ CFU/mL (9.43–9.49 log_10_ CFU/mL) among the five strains, while final CFS pH ranged from 4.0 to 4.1. The 24-h sampling point was selected based on previous work showing that CFS obtained after this cultivation period exhibited antimicrobial activity against foodborne pathogens (Tenea et al., 2025). Following cultivation, bacterial cells were removed by centrifugation at 13,000 × *g* for 30 min at 4 °C (Tenea et al., 2025). The resulting supernatants were passed through sterile 0.22-μm syringe filters (STF020025H, Chemlab Group, Washington, DC, USA), immediately frozen, lyophilized, and stored until metabolomic analysis. An uninoculated MRS medium control was incubated in parallel with the bacterial cultures at 37 °C for 24 h and subsequently subjected to the same centrifugation, filtration, freezing, lyophilization, extraction, and LC–MS/MS analytical workflow as the CFS samples. This incubated uninoculated MRS control was used to characterize the chemical background associated with the cultivation medium and incubation conditions. The MRS dataset was used as a background reference and was not mathematically subtracted from the CFS datasets.

### 2.2 Untargeted metabolome analysis

Untargeted metabolomic profiling was performed by ultra-performance liquid chromatography coupled with tandem mass spectrometry (UPLC-MS/MS; Metware Biotechnology Co., Ltd., USA). Lyophilized CFS samples from the five biological groups (G1–G5; BM1–BM15) were used. All biological replicates were processed independently throughout sample preparation and instrumental analysis. The analytical workflow included reverse-phase T3 and hydrophilic interaction liquid chromatography (HILIC) separations combined with electrospray ionization in positive and negative modes. Details of reagents, columns, solvents, and instrumentation are provided in Tables S2, S3.

#### 2.2.1 Sample preparation and extraction

Samples stored at −80 °C were thawed on ice before extraction. An aliquot of 150 μL extraction solvent consisting of acetonitrile:methanol (1:4, v/v) and containing internal standards was added to each sample (50 mg lyophilized powder). Samples were vortexed for 3 min and centrifuged at 12,000 rpm for 10 min at 4 °C. Subsequently, 150 μL of the supernatant was transferred to a new tube and incubated at −20 °C for 30 min to enhance protein precipitation. Samples were then centrifuged at 12,000 rpm for 3 min at 4 °C, and 120 μL of the resulting supernatant was transferred to LC-MS vials.

#### 2.2.2 Liquid chromatography conditions

##### 2.2.2.1 Reverse-phase (T3) chromatography

Metabolites were analyzed using an ExionLC AD UHPLC system (SCIEX, California, USA) coupled to an AB SCIEX TripleTOF 6600+ mass spectrometer (SCIEX, California, USA). Reverse-phase chromatographic separation was performed using a Waters ACQUITY UPLC HSS T3 column (1.8 μm, 2.1 × 100 mm). Mobile phase A consisted of ultrapure water containing 0.1% formic acid, and mobile phase B consisted of acetonitrile containing 0.1% formic acid (Table S4). The column temperature was maintained at 40 °C, the flow rate was 0.40 mL/min, and the injection volume was 4 μL. The gradient program was as follows: 0.0 min, 95% A; 2.0 min, 80% A; 5.0 min, 40% A; 6.0–7.5 min, 1% A; 7.6 min, 95% A; and 10.0 min, 95% A.

##### 2.2.2.2 Hydrophilic interaction liquid chromatography (HILIC)

HILIC separation was performed using an ACQUITY Premier BEH Amide column (1.7 μm, 2.1 × 150 mm). Mobile phase A consisted of 60% acetonitrile, 30% water, and 10% methanol containing 20 mM ammonium formate (pH 10.6), whereas mobile phase B consisted of 40% acetonitrile and 60% water containing 20 mM ammonium formate (pH 10.6). The column temperature was maintained at 40 °C, the flow rate was 0.40 mL/min, and the injection volume was 4 μL. The gradient program was as follows: 0.00 min, 95% A; 3.50 min, 70% A; 5.50–6.50 min, 5% A; 6.51 min, 95% A; and 10.00 min, 95% A.

### 2.3 Mass spectrometry conditions

Mass spectrometric analysis was performed using an AB SCIEX TripleTOF 6600+ mass spectrometer operated in both positive (ESI+) and negative (ESI–) electrospray ionization modes (Table S5). The ion spray voltage was set to 5,000 V in ESI+ mode and −4,000 V in ESI– mode. Source temperatures were 550 °C and 450 °C for ESI+ and ESI–, respectively. Ion source gases 1 and 2 were maintained at 50 and 60 psi, respectively, and curtain gas was set at 35 psi. The declustering potential was 60 V in ESI+ mode and −60 V in ESI– mode. MS1 and MS2 collision energies were set to 10 and 30 V in ESI+ mode and −10 and −30 V in ESI– mode, respectively, with a collision energy spread of 15 V. Data were acquired over an m/z range of 50–1,000 for MS1 and 25–1,000 for MS2. Accumulation times were 0.2 s for MS1 and 0.04 s for MS2. Eighteen candidate ions were selected per cycle, with dynamic exclusion applied for 3 s after three occurrences.

### 2.4 Data processing and metabolite annotation

LC–MS data files were converted to mzML format using ProteoWizard (Kessner et al., 2008). Peak extraction, feature alignment, and retention-time correction were performed using XCMS (Smith et al., 2006). Features with more than 50% missing values within a sample group were filtered, while missing values in the retained dataset were imputed using k-nearest-neighbor (KNN) imputation when the proportion of missing values was below 50% and one-fifth of the minimum detected value when it exceeded 50%. Peak areas were subsequently corrected using support vector regression (SVR).

Metabolite annotation was performed against the MetwareBio in-house database, integrated public and prediction databases, and metDNA. Features with a comprehensive identification score >0.5 and a coefficient of variation (CV) < 0.30 across quality-control (QC) samples were retained. Metabolite assignments were classified into confidence Levels 1a, 1b, 2, and 3 according to the MetWare annotation workflow. Level 1a corresponded to high-level matching and Level 1b to moderate-level matching based on the Standard criterion together with precursor-ion mass spectrometry (MS1), retention time (RT), and tandem mass spectrometry (MS2). Level 2 and Level 3 corresponded to high- and moderate-level matching, respectively, based on MS1, RT, and MS2 without the Standard criterion.

For compound-level differential-abundance analyses, only the higher-confidence Level 1a and 1b annotations were retained. Duplicate compound assignments across analytical configurations were consolidated by retaining the annotation with the highest confidence level or, when confidence levels were equivalent, the feature with the lower QC CV. The resulting consolidated dataset therefore excluded lower-confidence annotations and duplicate compound assignments removed during consolidation. Because the processing output did not provide a validated feature-level classification of excluded detections as adducts, in-source fragments, or other feature types, no further classification of these detections was performed.

The incubated uninoculated MRS medium was processed and analyzed using the same workflow and served as the culture-medium background control. It was not included as a biological group in the statistical comparisons among CFS samples. Higher-confidence compounds detected in CFS were compared with those detected in the MRS control as described in Section 2.6.4. Detection of a compound in CFS was considered part of the extracellular chemical profile and was not, by itself, interpreted as evidence of *de novo* bacterial biosynthesis.

### 2.5 Quality control and analytical reproducibility

A pooled quality-control (QC) sample was prepared by combining equal aliquots of all extracts and injected after every ten analytical samples to monitor instrument stability during acquisition. QC performance was evaluated by comparing total ion chromatograms (Figure S2), Pearson correlation among QC samples (Figure S3), and the distribution of coefficients of variation (CV) across detected metabolites (Figure S4). These assessments were used to evaluate analytical stability and reproducibility during LC-MS/MS acquisition. QC samples were not included in biological comparisons but were used exclusively to monitor analytical reproducibility throughout LC-MS/MS acquisition.

### 2.6 Statistical analysis

The metabolomics dataset comprised 15 biological samples (BM1–BM15), representing three independently cultured biological replicates for each of the five strains (G1–G5). Analyses of global metabolic profiles were performed independently for each analytical configuration using the corresponding QC-filtered feature matrix. Compound-level analyses were restricted to consolidated higher-confidence metabolite annotations (levels 1a and 1b), as described in Section 2.4. Abundance values were log_2_-transformed prior to statistical analysis.

#### 2.6.1 Principal component analysis (PCA)

PCA was used to examine global variation in extracellular chemical profiles and the distribution of biological replicates. PCA was performed independently for RP-T3 ESI+, RP-T3 ESI–, and HILIC ESI– using the complete QC-filtered feature matrix for each analytical configuration. QC samples were included in the score plots to assess analytical clustering. Covariance ellipses were included as graphical representations of within-group dispersion.

#### 2.6.2 Overall comparison and heatmap visualization

For the overall comparison among the five strains, relative abundance values for the 999 consolidated higher-confidence metabolite annotations were log_2_-transformed prior to statistical analysis. Normality was assessed using the Shapiro–Wilk test on model residuals, and homogeneity of variance was evaluated using Levene's test. Because unequal variances were observed for a substantial proportion of the annotations, Welch's one-way analysis of variance (Welch's ANOVA), which does not assume equal variances among groups, was used to compare the five strains. Resulting *P* values were adjusted for multiple testing using the Benjamini–Hochberg procedure, and annotations with FDR-adjusted *P* < 0.05 were considered significant. Annotations meeting this threshold were subsequently used for heatmap visualization of strain-associated abundance patterns.

#### 2.6.3 Differential metabolite analysis

Pairwise differential-abundance analyses were performed among the five strains (G1–G5) using consolidated higher-confidence metabolite annotations (levels 1a and 1b). For each of the ten pairwise comparisons, log_2_-transformed abundance values from the three independent biological replicates per strain were compared using Welch's two-sample *t*-test. Fold changes were calculated from group-level abundance values and expressed as log_2_ fold change (log_2_FC). To account for multiple testing, *P* values were adjusted using the Benjamini–Hochberg procedure. Annotations with BH-adjusted *P* < 0.05 and |log_2_FC| ≥ 1 were considered FDR-supported differential-abundance candidates. The UpSet representation was used to display the sizes of individual candidate sets and their exact intersections across comparisons.

#### 2.6.4 Medium-background comparison

Consolidated higher-confidence CFS metabolite annotations (MetWare Levels 1a and 1b) were compared with compounds detected in the incubated uninoculated MRS control. Exact-name correspondences were distinguished from possible matches based on molecular formula and retention-time proximity. This comparison was used to identify CFS annotations also detectable in the incubated culture-medium control and to provide context for interpretation of the extracellular chemical profiles (Table S6). The MRS control was not included as a biological group in statistical comparisons among strains.

### 2.7 Pathway annotation

KEGG pathway mapping was performed for higher-confidence metabolite annotations (MetWare Levels 1a and 1b) identified as differential-abundance candidates in the pairwise strain comparisons (BH-adjusted *P* < 0.05 and |log_2_FC| ≥ 1). Metabolites were mapped to their associated KEGG pathways, and the number of differential metabolites assigned to each pathway was determined for each pairwise comparison. Broad KEGG overview maps were excluded from visualization. Because individual metabolites may be associated with more than one KEGG pathway, the resulting pathway assignments were used to provide functional context for strain-associated differences and were not interpreted as evidence of pathway enrichment or pathway activity.

## 3 Results and discussion

### 3.1 Global metabolomic profiling and strain-associated variation

Untargeted LC–MS/MS provided broad coverage of the extracellular chemical profiles across the three analytical configurations. A total of 8,692 analytical detections were obtained, including 4,004 in RP-T3 ESI+, 2,574 in RP-T3 ESI–, and 2,114 in HILIC ESI–. Of these, 6,769 were associated with MS^2^ spectral information, comprising 3,060, 1,945, and 1,764 detections in RP-T3 ESI+, RP-T3 ESI–, and HILIC ESI–, respectively. The complementary chromatographic and ionization conditions expanded the chemical space represented in the CFS profiles. Amino acids and derivatives constituted the largest annotated chemical class (32.62%), followed by benzene derivatives (13.09%) and heterocyclic compounds (10.21%). Other represented classes included glycerophospholipids, glycerolipids, fatty acids, carbohydrates, organic acids, nucleobases, nucleotides, cofactors, alkaloids, and terpenoids (Figure S5). This diversity is consistent with the broad metabolic repertoire described for *L. plantarum*, particularly its capacities for carbohydrate utilization, amino acid metabolism, transport, and growth under diverse nutritional conditions (Papadimitriou et al., 2015; Siezen and van Hylckama Vlieg, 2011).

PCA revealed strain-associated variation across the three analytical configurations (Figure 1). In RP-T3 ESI+, PC1 and PC2 explained 28.98% and 17.06% of the total variance, respectively (46.04% combined). G1 and G5 formed relatively compact groups, whereas G2, G3, and G4 occupied different regions of the PCA space, with greater dispersion observed for G3 (Figure 1A). In RP-T3 ESI–, PC1 and PC2 accounted for 21.55% and 14.38% of the variance, respectively (35.93% combined). G1 was positioned predominantly in the negative PC1/positive PC2 region, G2 in the negative PC1/negative PC2 region, and G5 in the positive PC1/positive PC2 region. G3 and G4 were distributed mainly in the positive PC1/negative PC2 region, with differences in their relative positions along the two components (Figure 1B). In HILIC ESI–, PC1 and PC2 explained 20.74% and 14.21% of the variance, respectively (34.95% combined). G1 and G5 were located predominantly on the positive side of PC1, whereas G2 and G3 occupied different regions on the negative side, and G4 was positioned primarily toward negative PC2 values (Figure 1C). Biological replicates generally grouped according to strain, although within-group dispersion varied among strains and analytical configurations. QC samples clustered closely in all three datasets, supporting analytical reproducibility.

**Figure 1:**
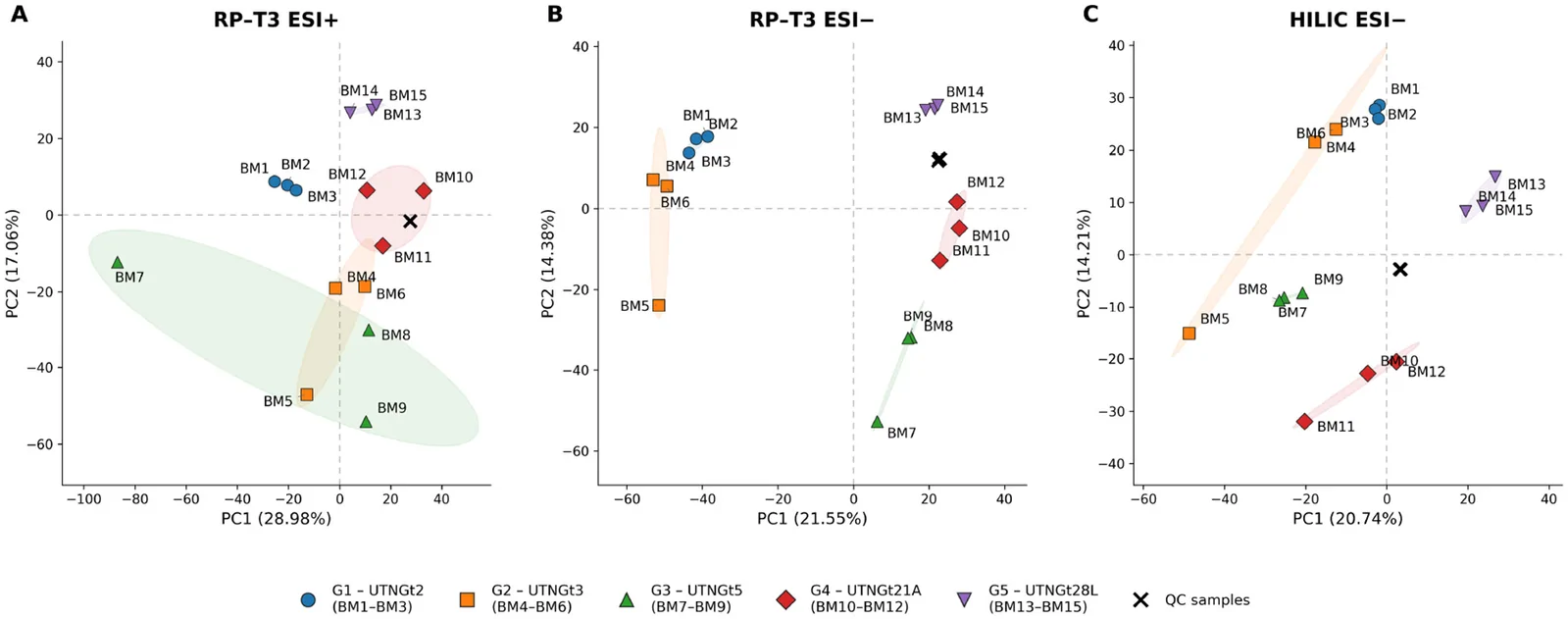
PCA of extracellular metabolomic profiles of five *L. plantarum* strains. PCA score plots were generated independently for **(A)** RP-T3 ESI+, **(B)** RP-T3 ESI–, and **(C)** HILIC ESI–datasets. G1–G5 correspond to UTNGt2 (BM1–BM3), UTNGt3 (BM4–BM6), UTNGt5 (BM7–BM9), UTNGt21A (BM10–BM12), and UTNGt28L (BM13–BM15), respectively, with three independent biological replicates per strain. Shaded ellipses illustrate within-group dispersion, and QC samples are indicated by black crosses. Percentages on the axes indicate the variance explained by the corresponding principal components.

The differences in PCA structure among RP-T3 ESI+, RP-T3 ESI–, and HILIC ESI– reflect the complementary selectivity of the analytical configurations, which capture compounds with different physicochemical and ionization properties. Overall, the profiles showed strain-associated structure without a uniform separation pattern across analytical conditions. Similar strain-dependent metabolomic variability has been reported for *L. plantarum* (Kim et al., 2023a,b) and other probiotic bacteria (Liu K. et al., 2025), supporting the relevance of strain-level characterization of bacterial metabolic phenotypes. Although biological replicates generally showed strain-associated grouping, the degree of within-group dispersion differed among strains and analytical configurations. This variability indicates that the extracellular chemical profiles were not uniformly reproducible at the individual-metabolite level across all biological cultures. The close clustering of QC samples supports analytical stability but does not establish the biological origin of within-group differences. Accordingly, PCA separation was interpreted as evidence of strain-associated variation rather than absolute strain-specific metabolic signatures. Besides, UTNGt2 (G1) and UTNGt5 (G3) originated from the same fruit species, *Theobroma grandiflorum*, yet exhibited 39 FDR-supported differential-abundance annotations. This observation suggests that a shared isolation host does not necessarily result in identical extracellular chemical profiles under standardized cultivation conditions. However, the present experimental design does not allow these differences to be attributed to host-associated adaptation.

At the 24-h CFS collection point, viable-cell densities ranged from 2.72 × 10^9^ to 3.08 × 10^9^ CFU/mL (9.43–9.49 log_10_ CFU/mL), with final pH values of 4.0–4.1 (Figure S1). Among the 999 consolidated higher-confidence CFS annotations, comparison with the incubated uninoculated MRS control identified 23 (2.30%) exact-name matches and five additional possible correspondences based on molecular formula and retention-time proximity (Table S6). Thus, 28 of 999 annotations (2.80%) showed either exact or possible correspondence with compounds detected in the MRS control. Compounds shared between CFS and MRS may represent residual medium components or compounds whose abundance was modified during bacterial growth through substrate utilization, transformation, release, or accumulation.

### 3.2 Pairwise differential-abundance patterns among strains

Pairwise differential-abundance analysis was performed using 999 consolidated higher-confidence metabolite annotations (levels 1a and 1b). The number of metabolites meeting both BH-FDR < 0.05 and |log_2_FC| ≥ 1 varied considerably among the ten pairwise comparisons (Figure 2). Four FDR-supported candidates were identified for G1 vs. G2, 39 for G1 vs. G3, 62 for G1 vs. G4, and 18 for G1 vs. G5. The remaining comparisons yielded 13 candidates for G2 vs. G3, 18 for G2 vs. G4, 19 for G2 vs. G5, 4 for G3 vs. G4, 53 for G3 vs. G5, and 48 for G4 vs. G5. The largest differential sets were observed for G1 vs. G4, G3 vs. G5, and G4 vs. G5, whereas G1 vs. G2 and G3 vs. G4 showed relatively few FDR-supported differences. Thus, the extent of extracellular chemical differentiation varied markedly among strain pairs. This pattern complements the global structure observed by PCA (Figure 1), in which the relative positions and dispersion of the strains also differed across analytical configurations. Additional metabolites met the nominal *P* < 0.05 and |log_2_FC| ≥ 1 criteria but did not remain significant after Benjamini–Hochberg correction (Figure 2). These candidates were retained as exploratory observations, whereas subsequent comparisons focused primarily on the FDR-supported metabolite set. The variation in the number of differential metabolites among strain pairs is consistent with the strain-dependent metabolomic heterogeneity previously reported for *L. plantarum* (Kim et al., 2023a,b).

**Figure 2:**
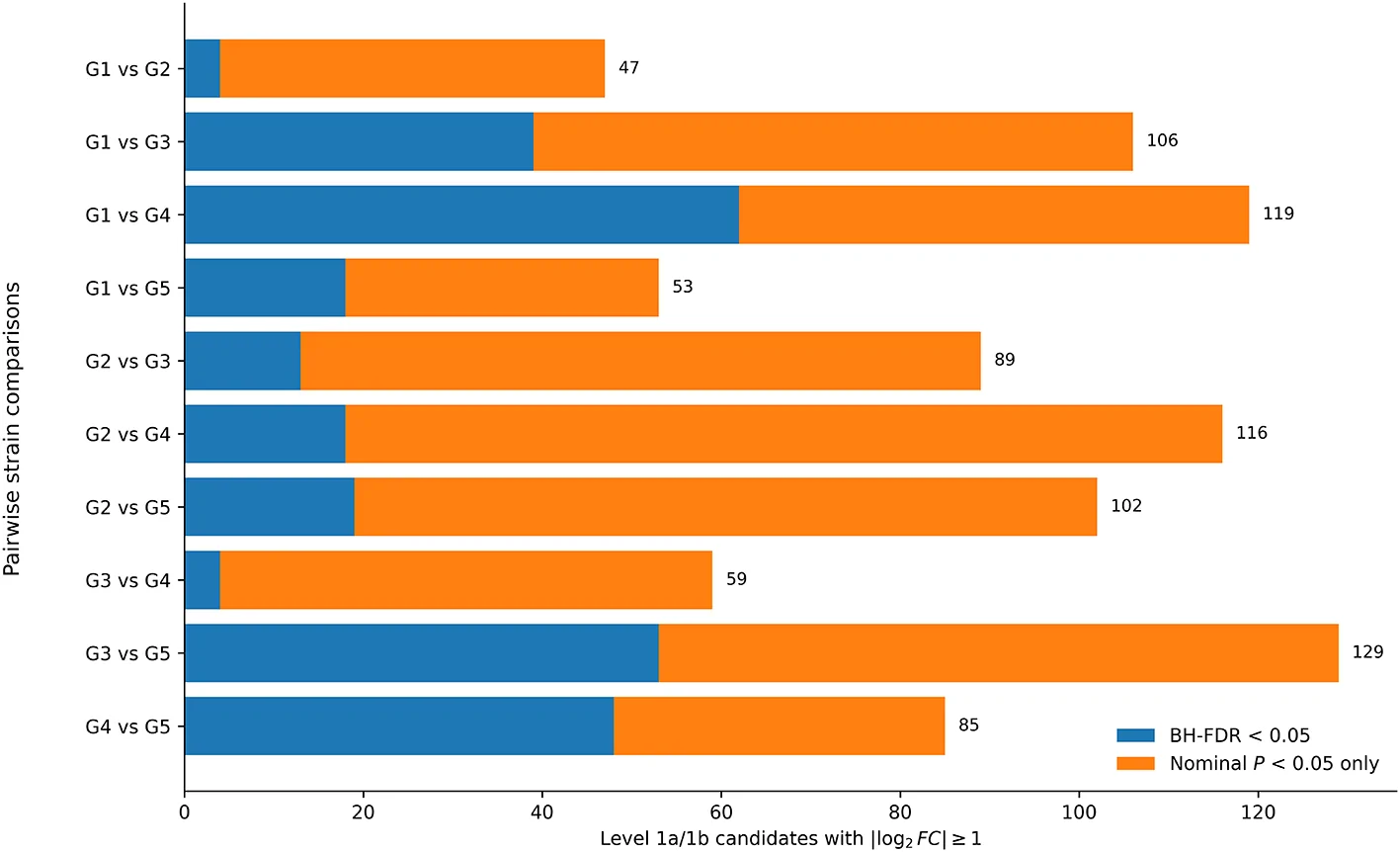
Pairwise differential-abundance analysis of higher-confidence metabolite annotations. Dark bars indicate annotations meeting both BH-FDR < 0.05 and |log_2_FC| ≥ 1, whereas light bars indicate exploratory annotations meeting nominal *P* < 0.05 and |log_2_FC| ≥ 1 but not the FDR threshold. G1, UTNGt2; G2, UTNGt3; G3, UTNGt5; G4, UTNGt21A; G5, UTNGt28L. Each group comprised three independent biological replicates.

### 3.3 Higher-confidence metabolites show distinct strain-associated abundance patterns

An overall comparison of the 999 consolidated higher-confidence annotations identified 438 annotations with significant differences among the five strains after Welch's one-way ANOVA and Benjamini–Hochberg correction (FDR < 0.05). The 30 annotations with the lowest adjusted *P* values were selected to visualize abundance patterns across the 15 independent biological samples (Figure 3). The heatmap revealed distinct strain-associated profiles involving compounds from several chemical classes. Among the most strongly differentiated annotations were flavin adenine dinucleotide, ketoleucine, N-acetylneuraminic acid, tagatose, erythrose, L-serine, *p*-aminobenzoic acid, guanosine-5′-monophosphate, 5-methoxytryptophol, D-phenylalanine, asparagine, histamine, indole-3-lactic acid, N-acetyl-β-alanine, and xylose (Figure 3). The three independent biological cultures generally showed similar abundance tendencies within each strain, although the magnitude of within-strain variation differed among metabolites.

**Figure 3:**
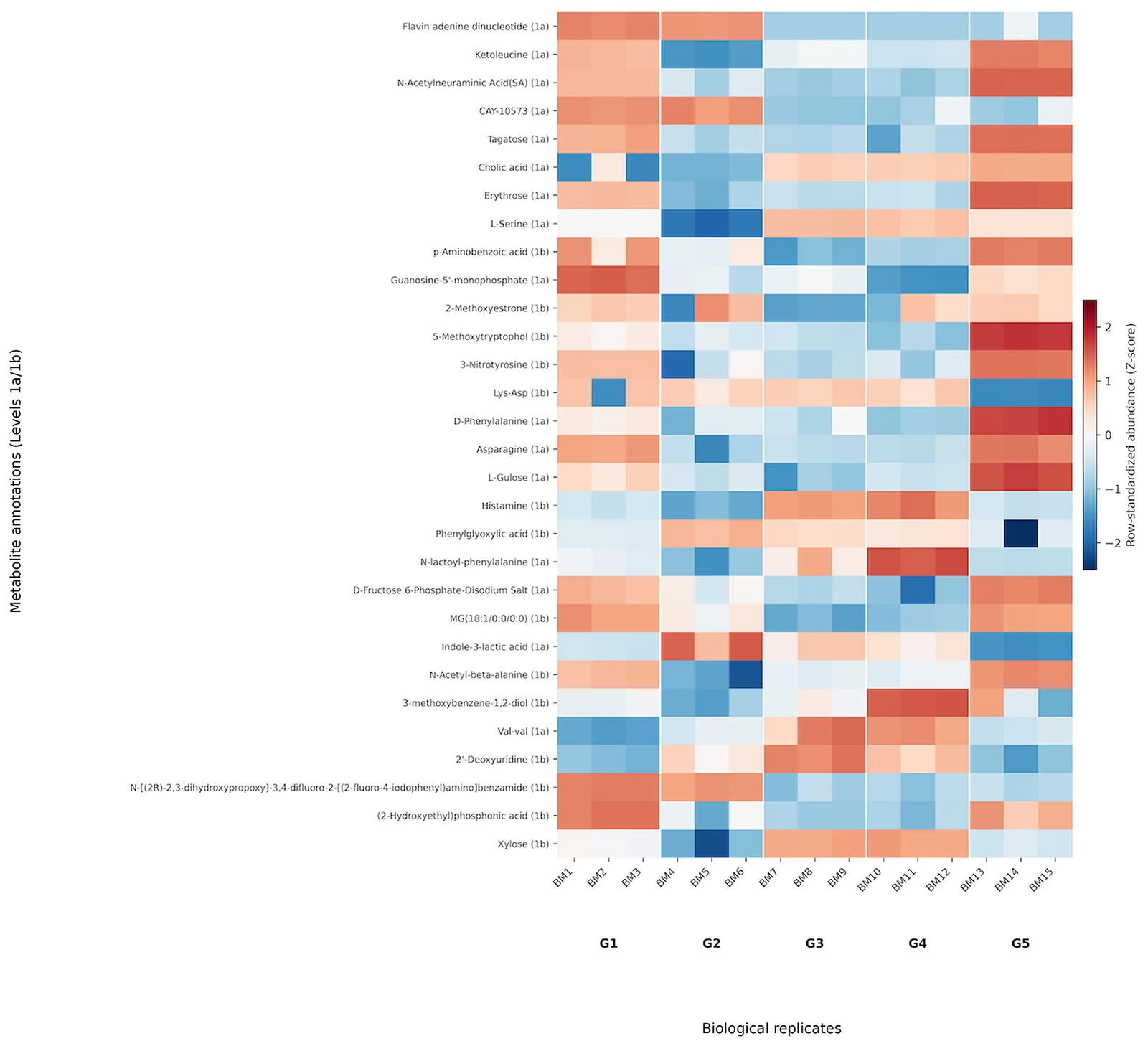
Heatmap of higher-confidence metabolite annotations showing significant differences among the five *L. plantarum* strains. The heatmap displays the 30 metabolite annotations with the lowest FDR-adjusted *P* values among the 438 annotations identified as significantly different among strains by Welch's one-way ANOVA followed by Benjamini–Hochberg correction (FDR < 0.05). Relative abundance values were log_2_-transformed and standardized by metabolite (Z-score) for visualization. Columns represent three independent biological cultures for each strain (G1–G5), and rows represent individual metabolite annotations. Annotation confidence levels (1a or 1b) are indicated in parentheses.

Several contrasting strain-associated patterns were apparent. L-serine showed markedly lower relative abundance in G2 than in the other groups, whereas erythrose, tagatose, and 5-methoxytryptophol showed comparatively higher abundance in G5. Histamine showed higher relative abundance in G3 and G4 and lower abundance in G2. Flavin adenine dinucleotide showed higher relative abundance in G1 and G2 than in G3–G5, whereas guanosine-5′-monophosphate was comparatively enriched in G1 and depleted in G4. Additional contrasting patterns were observed for ketoleucine, asparagine, indole-3-lactic acid, N-acetyl-β-alanine, and xylose. Together, these profiles demonstrate that the significant overall differences were not driven by a single uniform abundance pattern but involved distinct annotation-specific abundance patterns among strains.

The diversity of these abundance patterns is consistent with the metabolic flexibility described for *L. plantarum*. Strain-level differences in carbohydrate utilization, amino acid metabolism, transport systems, and regulatory functions have been reported for *L. plantarum* and provide relevant biological context for the chemical heterogeneity observed among strains (Papadimitriou et al., 2015; Siezen and van Hylckama Vlieg, 2011). The higher-confidence annotations highlighted in Figure 3 therefore provide specific candidates for subsequent targeted quantitative and functional investigation.

### 3.4 Distribution and overlap of differential metabolite sets

The UpSet analysis examined the distribution of FDR-supported higher-confidence metabolites across the four comparisons centered on G1 (Figure 4). The comparison sets comprised 4 candidates for G1 vs. G2, 39 for G1 vs. G3, 62 for G1 vs. G4, and 18 for G1 vs. G5. No metabolite annotation met the combined FDR and fold-change criteria in all four comparisons, and most candidates were restricted to individual comparisons or a limited subset of comparison sets. Consistent with the pairwise differential-abundance results (Figure 2), G1 differed only modestly from G2 but showed substantially larger differential sets relative to G3 and particularly G4. Together, these findings indicate that strain-associated extracellular differentiation involved comparison-specific combinations of metabolite annotations rather than a common discriminatory signature.

**Figure 4:**
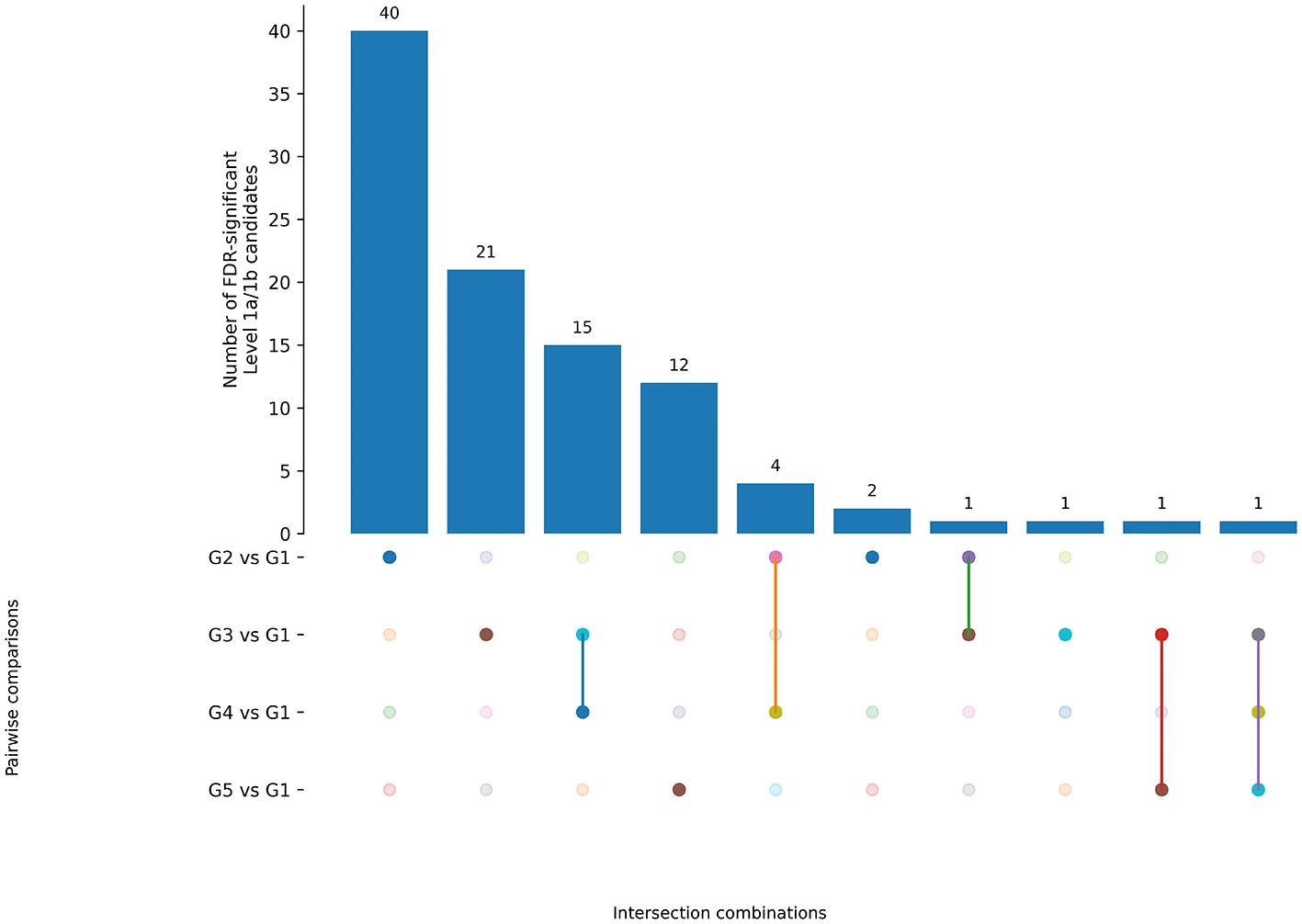
Overlap of FDR-supported higher-confidence metabolite annotations among G1-centered pairwise comparisons. The UpSet plot shows the distribution and exact intersections of annotations meeting the differential-abundance criteria (BH-FDR < 0.05 and |log_2_FC| ≥ 1) in G1 vs. G2, G1 vs. G3, G1 vs. G4, and G1 vs. G5. Vertical bars indicate the number of annotations in each exact intersection, while the connected-dot matrix identifies the comparisons contributing to each intersection. G1, UTNGt2; G2, UTNGt3; G3, UTNGt5; G4, UTNGt21A; G5, UTNGt28L. Each group comprised three independent biological replicates.

This pattern is consistent with the pronounced intraspecies metabolic diversity reported for *L. plantarum*. Comparative metabolomic profiling of 20 *L. plantarum* strains cultivated under common conditions identified distinct combinations of amino acids, peptides, purines, pyrimidines, alcohols, 2-hydroxy acids, and lipid-related compounds (Kim et al., 2023a). Strain-associated differences have also been reported in the extracellular metabolome of fruit-derived *L. plantarum*, including variation in amino acid-, carbohydrate-, and cofactor-related metabolic pathways (Tenea et al., 2026), while temporal profiling of the *L. plantarum* exometabolome demonstrated substantial changes in extracellular amino acid-, aromatic amino acid-, branched-chain amino acid-, and fatty acid-related metabolites during bacterial growth (Kim et al., 2023b).

The present study extends these observations through a direct side-by-side comparison of five fruit-derived *L. plantarum* strains cultivated and analyzed under the same experimental conditions. Unlike our previous strain-focused investigations, which addressed individual strains or different biological questions, the present design specifically evaluates the extent and distribution of extracellular chemical variation across multiple strains. The comparison-specific patterns observed here therefore provide information that could not be inferred from the previous studies alone and further support characterization at the individual-strain level when examining extracellular metabolic phenotypes (Kim et al., 2023a; Tenea et al., 2026).

### 3.5 Functional context of strain-associated metabolic variation

KEGG pathway mapping of the higher-confidence differential annotations provided functional context for the strain-associated variation observed in the extracellular profiles (Figure S6). The mapped metabolites were distributed across pathways related to amino acid, carbohydrate, nucleotide, lipid, and cofactor metabolism, although their representation differed considerably among pairwise comparisons. This comparison-dependent distribution is consistent with the differential-abundance analysis (Figure 2) and the limited overlap among metabolite sets observed in the UpSet analysis (Figure 4), indicating that extracellular differentiation among the strains involved different combinations of metabolic functions.

Amino acid-related pathways were recurrently represented across the comparisons, including tyrosine metabolism, histidine metabolism, D-amino acid metabolism, and valine, leucine, and isoleucine biosynthesis and degradation (Figure S6). These pathway associations complement the metabolite-level patterns shown in Figure 3, where several amino acid- and peptide-related annotations, including L-serine, ketoleucine, D-phenylalanine, asparagine, histamine, Lys-Asp, N-lactoyl-phenylalanine, and Val-Val, were among the annotations showing the strongest overall differences among strains. The prominence of this chemical group is consistent with the extensive amino acid transport and metabolic capabilities of *L. plantarum*, which contribute to nitrogen utilization, stress adaptation, and metabolic flexibility (Papadimitriou et al., 2015; Siezen and van Hylckama Vlieg, 2011).

Carbohydrate-associated variation was also evident, with galactose metabolism represented across several pairwise comparisons (Figure S6). This observation is consistent with the occurrence of carbohydrate-related annotations among the strongly differentiated compounds shown in Figure 3, including erythrose, tagatose, L-gulose, D-fructose 6-phosphate disodium salt, and xylose. The capacity of *L. plantarum* to utilize a broad range of carbohydrates is a characteristic feature of its metabolic versatility, and considerable strain-level variation has been reported in carbohydrate transport and utilization systems (Siezen and van Hylckama Vlieg, 2011; Cui et al., 2021). Such variability provides relevant biological context for the strain-associated differences in extracellular chemical profiles observed under the same nutritional conditions.

Nucleotide metabolism showed particularly marked comparison-dependent representation. Pyrimidine metabolism was most strongly represented in G3 vs. G5 and G4 vs. G5, while purine metabolism was also prominent in G4 vs. G5 (Figure S6). These results are consistent with the presence of nucleotide-related annotations among the differentiated compounds, including guanosine-5′-monophosphate in Figure 3. Lipid-associated pathways, including glycerophospholipid metabolism and fatty acid biosynthesis, were also represented in several comparisons, together with cofactor-related pathways such as lipoic acid metabolism. The representation of these metabolite classes indicates that the strain-associated differences in extracellular chemical profiles extended beyond carbohydrate- and amino acid-related annotations. Overall, KEGG mapping of the higher-confidence differential annotations provided descriptive functional context for the observed strain-associated differences. This agrees with previous metabolomic studies demonstrating substantial strain-dependent variation in *L. plantarum* involving multiple metabolite classes rather than a single conserved metabolic signature (Kim et al., 2023a; Tenea et al., 2026). Because individual metabolites can be assigned to multiple KEGG pathways, the associations shown in Figure S6 should be considered functional context for the differential metabolite profiles rather than evidence of pathway activation or enrichment. Their distribution across comparisons identifies amino acid, carbohydrate, nucleotide, lipid, and cofactor metabolism as areas for subsequent targeted investigation.

### 3.6 Study limitations

Several factors should be considered when interpreting the differences in extracellular chemical profiles among strains. Metabolite abundance was not normalized to biomass or cellular protein; therefore, differences in extracellular abundance may reflect variation in metabolic activity relative to biomass, in addition to strain-associated metabolic characteristics. Viable-cell densities and final pH showed relatively narrow ranges among strains at the 24-h CFS collection point, indicating broadly comparable culture conditions at the time of sampling. However, CFU counts represent culturable viable cells rather than total cellular biomass and therefore cannot replace direct biomass or protein normalization. Future studies incorporating biomass measurements or cellular protein content would allow extracellular metabolite abundance to be evaluated relative to culture biomass.

The detection of nucleotide-related and other intracellular-associated compounds in CFS does not necessarily indicate active secretion. Their presence may result from transport, release during cultivation, or cellular disruption, in addition to contributions from the complex culture medium. Although bacterial cells were removed by centrifugation and filtration before metabolomic analysis, these procedures do not establish whether soluble compounds originated from intact or lysed cells. Cell integrity, autolysis, and biofilm formation were not directly assessed. Therefore, the mechanisms responsible for the extracellular occurrence of these compounds should be further investigated.

The use of complex MRS medium also limits direct attribution of individual extracellular compounds to bacterial biosynthesis. The chemical composition of the final CFS reflects the combined effects of residual medium components, substrate consumption and transformation, and metabolite release or accumulation during bacterial growth. Similar patterns have been reported in untargeted analyses of probiotic secretomes, where comparison with uninoculated medium showed both depletion and accumulation of specific compounds during cultivation (Lu et al., 2026). In the present study, incubated uninoculated MRS processed under the same conditions provided a background reference for compounds also detectable in the cultivation medium. Nevertheless, establishing the microbial origin of individual compounds would require additional experiments using defined media, isotope-tracing approaches, or targeted analytical methods.

Finally, the metabolite annotations and relative-abundance patterns identified here require targeted quantification and experimental validation. Activity-guided analysis of selected candidates would be necessary to determine whether specific extracellular compounds contribute to the previously observed antimicrobial activity of the corresponding CFS. Such studies could help identify chemical markers associated with antimicrobial activity and support the future characterization and standardization of bioactive CFS preparations.

## 4 Conclusions

The extracellular chemical profiles of the five fruit-derived *L. plantarum* strains showed substantial strain-associated variation despite cultivation under the same experimental conditions. Differences were evident in both the global LC–MS/MS profiles and the higher-confidence metabolite annotations, with the degree of differentiation varying among strain pairs. The chemical variation involved metabolites from several classes, including amino acids and derivatives, carbohydrates, organic acids, nucleotides, lipids, and cofactors. KEGG mapping further indicated that these differences were associated with multiple metabolic functions, particularly amino acid, carbohydrate, nucleotide, lipid, and cofactor metabolism.

The differential metabolite annotations identified in this study provide a basis for further investigation of the extracellular chemical composition of these strains and its possible relationship with the previously demonstrated antimicrobial activity of their CFS. Targeted quantification and activity-guided studies will be needed to determine whether specific metabolites, or combinations of metabolites, are associated with differences in inhibitory activity among CFS. Further characterization of these relationships may help identify chemical markers associated with antimicrobial activity and support the selection and standardization of fruit-derived *L. plantarum* CFS for future antimicrobial applications.

## Data Availability

The original contributions presented in the study are included in the article/Supplementary material, further inquiries can be directed to the corresponding author.
