# Supplementary material for: Strain-associated variation in extracellular chemical profiles of fruit-derived Lactiplantibacillus plantarum under standardized cultivation
Source: Front Microbiol. 2026 Oct 30;17:1966445. doi: 10.3389/fmicb.2026.1966445 (PMC13760199; doi:10.3389/fmicb.2026.1966445)
Supplement: Supplementary file 1 [file Data_Sheet_1.zip › Supplementary materials.docx]

Table S1. Samples description

| **Species** | **Origin** | **Description samples** | **Sample code** | **Group** |
| --- | --- | --- | --- | --- |
| *Lactiplantibacillus plantarum* UTNGt2 | *Theobroma grandifolium* | CFS | BM1 | G1 |
|  |  |  | BM2 |  |
|  |  |  | BM3 |  |
| *Lactiplantibacillus plantarum* UTNGt3 | *Chrysophyllum oliviforme* | CFS | BM4 | G2 |
|  |  |  | BM5 |  |
|  |  |  | BM6 |  |
| *Lactiplantibacillus plantarum* UTNGt5 | *Theobroma grandifolium* | CFS | BM7 | G3 |
|  |  |  | BM8 |  |
|  |  |  | BM9 |  |
| *Lactiplantibacillus plantarum* UTNGt21A | *Solanum quitoense* | CFS | BM10 | G4 |
|  |  |  | BM11 |  |
|  |  |  | BM12 |  |
| *Lactiplantibacillus plantarum* UTNGt28L | *Chrysophyllum cainito* | CFS | BM13 | G5 |
|  |  |  | BM14 |  |
|  |  |  | BM15 |  |

Table S2. Information of standards and reagents

| **Compound** | **CAS** | **Purity** | **Brand** |
| --- | --- | --- | --- |
| Methanol | 67-56-1 | more than 99.9% | Thermo Fisher |
| Acetonitrile | 75-05-8 | 99.95% | Thermo Fisher |
| Acetic Acid | 64-19-7 | more than 99.7% | Thermo Fisher |
| Ammonium formate | 540-69-2 | more than 99.0% | Sigma |
| Ammonium hydroxide solution(25%) | 1336-21-6 | - | Sigma |
| Formic Acid | 64-18-6 | 97.5-98.5% | Sigma |
| Standard | - | more than 98% | isoreag/TRC/TCI/Sigma |

Table S3. Information of instruments

| **Name** | **Instrument** | **Brand** | **Address** |
| --- | --- | --- | --- |
| Mass Spectrometer | TripleTOF 6600+ | SCIEX | California, USA |
| UHPLC | ExionLC AD | SCIEX | California, USA |
| Centrifuge | 5430R | Eppendorf | Hamburg, Germany |
| Vortex mixer | MI0101002 | HFour E's | Guangzhou, China |
| Electronic balance(1/100000) | AS60/220.R2 Plus | Radwag | Poland |
| Centrifugal concentrator | CentriVap | LABCONCO | Missouri Kansas, USA |
| Ultrasonic cleaner | CD-F15 | Olenyer | China |
| Pipette | Research plus | Eppendorf | Hamburg,Germany |
| Automatic workstation | Biomek i5 | Beckman Coulter | California, USA |
| Uniform-Seal Heat Sealer | FM 5200 | foodsaver | California, USA |

Table S4. Elution gradient for T3

| **Time (min)** | **A (%)** | **B (%)** |
| --- | --- | --- |
| 0 | 95 | 5 |
| 2 | 80 | 20 |
| 5 | 40 | 60 |
| 6 | 1 | 99 |
| 7.5 | 1 | 99 |
| 7.6 | 95 | 5 |
| 10 | 95 | 5 |

Table S5. Mass spectrometry acquisition parameters for positive- and negative-ion electrospray modes.

| Parameter | ESI+ | ESI- |
| --- | --- | --- |
| Duration (min) | 10 | 10 |
| IonSpray Voltage (V) | 5000 | -4000 |
| Temperature (°C) | 550 | 450 |
| Ion Source Gas1 (psi) | 50 | 50 |
| Ion Source Gas2 (psi) | 60 | 60 |
| Curtain Gas (psi) | 35 | 35 |
| Declustering Potential (V) | 60 | -60 |
| MS1 Collision Energy (V) | 10 | -10 |
| MS2 Collision Energy (V) | 30 | -30 |
| Collision Energy Spread (V) | 15 | 15 |
| MS1 TOF Masses (Da) | 50~1000 | 50~1000 |
| MS2 TOF Masses (Da) | 25~1000 | 25~1000 |
| MS1 Accumulation time (s) | 0.2s | 0.2s |
| MS2 Accumulation time (s) | 0.04s | 0.04s |
| Candidate ions | 18 | 18 |
| Exclude former target ions | Always, For 3 seconds, After 3 occurences | Always, For 3 seconds, After 3 occurences |

**Table S6**. Conservative correspondence between higher-confidence CFS metabolite annotations and compounds detected in uninoculated MRS medium.

| **Feature ID** | **CFS annotation** | **MetWare level** | **Molecular formula** | **CFS analytical mode** | **CFS RT (min)** | **MRS correspondence** | **MRS annotation** | **MRS annotation level** |
| --- | --- | --- | --- | --- | --- | --- | --- | --- |
| MW0104770 | 2-Hydroxycinnamic acid | 1a | C9H8O3 | T3_positive | 1.2679 | Exact-name overlap | 2-Hydroxycinnamic acid | 2 |
| MW0168601 | 4-Hydroxybenzaldehyde | 1a | C7H6O2 | T3_negative | 3.6624 | Exact-name overlap | 4-Hydroxybenzaldehyde | 2 |
| MW0105438 | Adenine | 1a | C5H5N5 | HILIC_negative | 1.2701 | Exact-name overlap | Adenine | 2 |
| MW0168692 | Adenosine | 1b | C10H13N5O4 | T3_positive | 2.2039 | Exact-name overlap | Adenosine | 2 |
| MW0105654 | Arg-Glu | 1b | C11H21N5O5 | T3_positive | 1.7702 | Exact-name overlap | Arg Glu | 3 |
| MW0017091 | Cholic acid | 1a | C24H40O5 | T3_negative | 6.0003 | Exact-name overlap | Cholic acid | 2 |
| MEDN0304 | Cinnamic Acid | 1a | C9H8O2 | HILIC_negative | 1.053 | Exact-name overlap | Cinnamic acid | 3 |
| MW0106168 | Citric acid | 1a | C6H8O7 | T3_negative | 1.0142 | Exact-name overlap | Citric acid | 2 |
| MW0053754 | Glycocholic acid | 1b | C26H43NO6 | T3_positive | 6.3852 | Exact-name overlap | Glycocholic acid | 2 |
| MW0103577 | Guanosine | 1b | C10H13N5O5 | T3_positive | 1.3239 | Exact-name overlap | Guanosine | 2 |
| MW0159863 | Hypoxanthine | 1a | C5H4N4O | HILIC_negative | 1.359 | Exact-name overlap | Hypoxanthine | 2 |
| MW0107537 | Indole | 1a | C8H7N | T3_positive | 2.4212 | Exact-name overlap | Indole | 2 |
| MW0159913 | Inosine | 1b | C10H12N4O5 | T3_negative | 6.2137 | Exact-name overlap | Inosine | 2 |
| MW0153298 | Maculosin | 1b | C14H16N2O3 | T3_negative | 2.8801 | Exact-name overlap | Maculosin | 2 |
| MW0108213 | Methionine | 1a | C5H11NO2S | HILIC_negative | 1.9863 | Exact-name overlap | Methionine | 2 |
| MEDP1577 | Norvaline | 1a | C5H11NO2 | HILIC_negative | 2.1481 | Exact-name overlap | Norvaline | 3 |
| MW0009415 | Phenylacetic acid | 1b | C8H8O2 | T3_negative | 2.5656 | Exact-name overlap | Phenylacetic acid | 2 |
| MW0109274 | Pipecolic acid | 1a | C6H11NO2 | T3_positive | 0.712 | Exact-name overlap | Pipecolic acid | 2 |
| MEDL00098 | Pseudouridine | 1b | C9H12N2O6 | HILIC_negative | 3.7585 | Exact-name overlap | Pseudouridine | 3 |
| MW0107948 | Pyroglutamic acid | 1b | C5H7NO3 | HILIC_negative | 3.513 | Exact-name overlap | Pyroglutamic acid | 3 |
| MW0169982 | Tyramine | 1a | C8H11NO | T3_positive | 1.2528 | Exact-name overlap | Tyramine | 3 |
| MW0110297 | Tyrosyl-Valine | 1b | C14H20N2O4 | HILIC_negative | 2.3606 | Exact-name overlap | Tyrosyl-Valine | 3 |
| MW0103709 | Uridine-5'-monophosphate | 1a | C9H13N2O9P | T3_negative | 1.3796 | Exact-name overlap | Uridine 5'-monophosphate | 2 |
| MW0103980 | (r)-3-Amino-4-methylpentanoic acid | 1b | C6H13NO2 | HILIC_negative | 1.1802 | Possible formula/RT match | Norleucine | 2 |
| MW0002155 | 2,3-Dihydrobenzofuran | 1a | C8H8O | T3_positive | 1.2528 | Possible formula/RT match | Phenylacetaldehyde | 2 |
| MW0133678 | 4-Hydroxycinnamic acid | 1b | C9H8O3 | T3_positive | 1.2591 | Possible formula/RT match | 2-Hydroxycinnamic acid | 2 |
| MEDP0510 | Carnitine C2:0 | 1b | C9H17NO4 | HILIC_negative | 1.162 | Possible formula/RT match | Acetylcarnitine | 3 |
| MEDP0519 | L-Norleucine | 1b | C6H13NO2 | T3_positive | 1.3239 | Possible formula/RT match | Isoleucine | 3 |

Abbreviations: ESI+, positive electrospray ionization; ESI−, negative electrospray ionization; MSI, Metabolomics Standards Initiative confidence level.
